# Odontome: A Brief Overview

**DOI:** 10.5005/jp-journals-10005-1106

**Published:** 2011-04-15

**Authors:** V Satish, Maganur C Prabhadevi, Rajesh Sharma

**Affiliations:** 1 Associate Professor, Department of Pedodontics and Preventive Dentistry, Jaipur Dental College, Jaipur, Rajasthan, India; 2 Reader, Department of Pedodontics and Preventive Dentistry, Jaipur Dental College, Jaipur, Rajasthan, India; 3 Professor, Department of Pedodontics and Preventive Dentistry, Jaipur Dental College, Jaipur, Rajasthan, India

**Keywords:** Odontomes, Complex and compound odontomes.

## Abstract

Odontomas are the most common type of odontogenic tumors. They are included under the benign calcified odontogenic tumors. Odontomas are basically classified into two types, complex and compound odontomes. Various theories or etiological factors are been quoted for the occurrence of odontomes. Generally, they are asymptomatic. Occasionally, signs and symptoms relating to their presence do occur. The sole management depends upon the early diagnosis, histopathological examination and excision of these tissues. This article briefs regarding its classification, etiological factors, occurrence, differences between complex and compound odontomes, diagnosis and management.

## INTRODUCTION

Odontome in medicine and dentistry was originally used for any tumor and/or tumor-like lesion, like neoplastic cyst arising from tooth forming tissues.^1^ Odontomas are hamartomas of aborted tooth formation which account for 22% of the odontogenic tumors.^2^ They are the most common benign odontogenic tumors of epithelial and mesenchymal origin.^3^ In 1974, Shafer, Hine and Levy^4^ described odontomes as tumors of odontogenic origin but their current views^5^ support that an odontome is now widely accepted by most authorities as a hamartoma.

The term odontome was coined by Paul Broca in 1867. Broca defined the term as tumors formed by the overgrowth or transitory of complete dental tissue.^6^ Odontomas by definition alone refers to any tumor of odontogenic origin. This is because odontomas result from the growth of completely differentiated epithelial and mesenchymal cells that give rise to ameloblasts and odontoblasts.^5^ In a broad sense, it means a growth with both the epithelial and mesenchymal components exhibiting complete differentiation resulting in functional ameloblasts and odontoblasts.^5^ These cells in turn form variable amounts of enamel and dentin and pulpal tissue of the odontoma.^7^ This enamel and dentin were usually laid down in an abnormal pattern because the organization of odontogenic cells failed to reach the normal state of mor-phodifferentiation.^5^ So they are considered as developmental anomalies rather than true neoplasm.

Odontomas constitute about 22% of all odontogenic tumors of the jaws.^2^ Approximately, 10% of all odontogenic tumors of the jaws are compound odontomas.^8,9^ The incidence of compound odontome ranges between 9 and 37% and the complex odontome is between 5 and 30%.^10^ Odontomas are discovered during the second and third decades of life.^3,7,8^ The compound odontoma is slightly more common than the complex odontoma which in turn is more common than the ameloblastic odontoma. The majority of odontomas in the anterior segment of the jaws are compound composite in type (61%), whereas the majority in the posterior segment is complex composite in type (34%).^5^ Interestingly both type of odontomas occurred more frequently on the right side of the jaw than on the left, (compound 62%, complex 68%).^5^ The compound composite odontome most frequently occurred in incisor cuspid region of the upper jaw in contrast to the complex odontome which were commonly found in molar and premolar region of the mandible.^1,11,12^ Some reports have reported presence of both the types of odontomes in different locations, such as maxillary sinus, according to Bland Sutton (1988)^1,13^ in which 300 denticles were seen bilaterally, mandibular ramus,^14^ subcondylar region^15^ or mental foramen,^13^ mid palate^16^ and the middle ear.^17^ Hermann (1957) reported a case of compound composite odontome which consisted 2,000 denticles.^1^

Odontomes commonly occur in permanent dentition (Table 1) and are rarely reported in association with primary teeth (Table 2).^18,19^ Association of odontomes with the deciduous dentition is rare. Tratman (1949)^20^ thought that the deciduous dentition was not prone to the formation of odontomes. Saeed and Khalid noted presence of multiple odontomas in both maxilla and mandible in a female aged 7 years.^20^ In the review done by Katz,^21^ only 5 (2%) of 396 odontomas were associated with failure of a primary tooth to erupt.

**Table 1:** **Table 1:** Reported cases of odontomas associated with permanent teeth (up to 15 years)

*Authors*		*Year*		*Age at* *which* *seen*		*Sex*		*Teeth associated with*		*Number*		*Relation* * within the* * bone*		*Type*	
Goldberg et al^63^		1981		14 years		F		Mandibular molar		Solitary		Intraosseous		Complex	
Torreti et al^26^		1983		12 years		F		Maxillary central incisor		Multiple		Intraosseous		Compound	
Smith^62^		1985		12 years		F		Maxillary molar		Multiple		Intraosseous		Complex/	
														compound	
Lopez Areal et al^14^		1992		12 years		F		Maxillary central incisor		Solitary		Intraosseous		Compound	
Kaneko et al^46^		1998		14 years		F		Mandibular molar		Solitary		Intraosseous		Complex	
Ajike et al^60^		2000		15 years		F		Maxilla and mandible		Multiple		Intraosseous/		Compound	
												extraosseous			
Oliveira et al^65^		2001		12 years		F		Maxilla		Multiple		Intraosseous		Compound	
Oliveira et al^65^		2001		11 years		M		Maxilla		Multiple		Intraosseous		Compound	
Batra et al^40^		2004		14 years		M		Maxillary central incisor		Solitary		Intraosseous		Complex	
Batra et al^40^		2004		14 years		M		Maxillary central incisor		Solitary		Intraosseous		Compound	
Amailuk and Grubor^61^		2008		15 years		M		Maxillary central incisor		Solitary		Extraosseous		Erupted compound	
														odontoma	
Usha Mohan Das et al^64^		2008		11 years		F		Maxillary central incisor		Solitary		Intraosseous		Compound	
														composite	
														odontoma	
Shekar et al^22^		2009		15 years		F		Mandibular molar		Solitary		Extraosseous		Erupted compound	
														odontome	

**Table 2:** **Table 2:** Reported cases of odontomas associated with deciduous teeth

*Authors*		*Year*		*Age at which seen*		*Sex*		*Teeth associated*		*Number*		*Relation within the bone*		*Type*	
Axel^50^		1937		4 years		M		Maxillary canine		Solitary		Intraosseous		Compound	
Hitchin and Dekonor^50^		1963		5 years		M		Maxillary canine		Solitary		Intraosseous		Compound	
Hitchin and Dekonor^50^		1963		8 years		F		Maxillary canine		Solitary		Intraosseous		Compound	
Schreiber^51^		1963		9 years		M		Bilat maxillary incisors		Multiple		Intraosseous		Complex	
Bader^52^		1967		5 years		F		Post parts of both jaws		Multiple		Intraosseous/		Ameloblastic	
												extraosseous		Fibroma/	
														compound	
Hunsuck^16^		1970		Infant		M		Mid palatal		Solitary		Intraosseous		Compound	
Noonan^53^		1971		5 years		F		Maxillary canine		Solitary		Intaosseous		Compound	
Saeed and Khalid^20^		1974		7 years		F		Both jaws		Multiple		Intraosseous		Complex/	
														compound	
Zordan Z Stajcic^19^		1987		6 years		M		Maxillary canine		Solitary		Intraosseous		Compound	
Motokawa et al^54^		1990		3 years		F		Maxillary molar		Solitary		Intraosseous		Complex	
Castro et al^55^		1994		6 years		M		Mandibular molar		Solitary		Extraosseous		Peripheral	
														odontomas	
Bacetti T^56^		1995		3.5 years		F		Maxillary canine		Multiple		Intraosseous		Compound	
Khuran et al		1997		1 year		M		Maxillary canine		Solitary		Intraosseous		Ameloblastic	
														fibro-odontoma	
Oliveira et al^65^		2001		5		F		Maxillary central incisor		Multiple		Intraosseous		Compound	
Yeung & cheung		2003		2 years		M		Maxillary central incisor		Multiple		Intraosseous		Compound	
& Tsang^57^				5 months											
Nelson- Filho P et al^58^		2005		1 year		F		Maxillary incisor		Solitary		Intraosseous		Compound	
				8 months											
Dror Aizenbud and		2008		5.5 years		M		Maxillary central incisor		Solitary		Intraosseous		Compound	
Yael Pery Front^59^															

## ETIOLOGY

The etiology behind odontomes remains unknown.^5^ It has been related to various pathological conditions, like local trauma, inflammatory and/or infectious processes, mature ameloblasts, cell rests of serres (dental lamina remnants) or due to hereditary anomalies (Gardner’s syndrome, Hermanns syndrome), odontoblastic hyperac-tivity, alterations in the genetic component responsible for controlling dental development.^22^ Hitchin suggested that odontomes are inherited or are due to a mutagene or interference, possibly postnatal, with the genetic control of tooth development.^23^

**Table 3:** **Table 3:** Differences between compound and complex odontomes

		*Compound odontome*		*Complex odontome*	
Definition		Malformation in which all dental tissues are represented in a more orderly pattern than in the complex odontome so that the lesion consists of many tooth-like structures		Malformation in which all dental tissues are represented, individual tissues being mainly well-formed cut occurring in more or less disorderly pattern	
Shape		Regularly shaped, solitary or multiple small denticles		Amorphous conglomeration of dental tissues	
Appearance		Bizzare peg shaped teeth show anatomic resemblance to normal teeth		An irregular mass No morphologic similarity	
Composition		They are formed of enamel and dentin, also have variable amounts of cementum and pulpal tissue		These tumors are formed of enamel and dentin, but they can also have variable amount of cementum and pulp tissue	
Incidence		9 to 37%		5 to 30%	
Sex		More common in females		It is seen more often in female patients	
		But they are also seen both in males and females			
Age		Commonly seen in second and third decades. Both complex and compound odontomas are most commonly found in younger patients with mean ages reported to be 14.8 and 20.3 years respectively.^48^			
Classification		Compound odontome was classified as acc to Gravey et al		It occurs as a single mass or conglomeration of tissues	
		• *Denticulo type* (Fig. 1): Composed of two or more separate denticles, having crown and root, dental hard tissues resembling to that of tooth			
		• *Particulate type* (Fig. 1): Composed of two or more separate masses or particles, bearing no resemblance to tooth, consists of hard dental tissues			
		• *Denticulo-particulate type:* In this type, denticles and particles are present together.		
		Commonly situated in the anterior segment (incisor-canine region of maxilla) of the jaws		Commonly situated in the molar region of mandible (first and second molar areas of the mandible)	
Site predilection		But also located in rare instances in maxillary sinuses, pituitary region, subcondylar region, ramus of the mandible, middle ear, mental foramen and midpalatal region.			
		Both type of odontomas occurred more frequently on the right side of the jaw than on the left.^49^			
Associated		Commonly occur in permanent dentition and are rarely reported in association with primary teeth^18-21^			
Signs and		• Asymptomatic, although occasionally signs and symptoms relating to their presence do occur			
Symptoms		• These generally consist of			
		– Unerupted or impacted teeth,			
		– Impacted tooth,			
		– Retained deciduous teeth,			
		– Swelling and evidence of infection,			
		– Displacement of teeth and malocclusion.			
		• Compound odontomes seldom cause bony expansion but complex odontome often cause slight or even marked bony expansion.			
Diagnosis		Both the lesion can be diagnosed by routine radiographs , but newer diagnostic techniques such as microradiography can be used.			
Radiological features		Comparatively well-organized malformed teeth or tooth-like structures or denticles of varying size and shape surrounded by a narrow radiolucent zone (Fig. 2).		Irregular mass of calcified material surrounded by a narrow radiolucent band with a smooth outer periphery (Fig. 3).	
Histopathology		Tooth-like structures with central cores of pulp tissue that are encased in shells of dentin and partially covered by enamel surrounded by a fibrous capsule similar to the follicle surrounding a normal tooth.		Microscopically they consist of haphazard conglomerates of dentin, enamel, enamel matrix, cementum and pulp tissue.	
Treatment		Surgical excision			

**Fig. 1:**
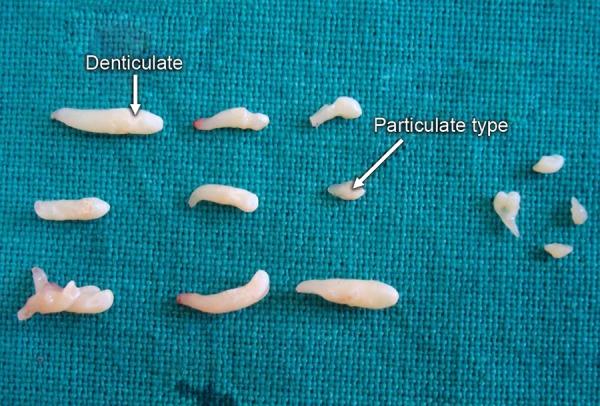
Denticulo and Particulate type

**Fig. 2:**
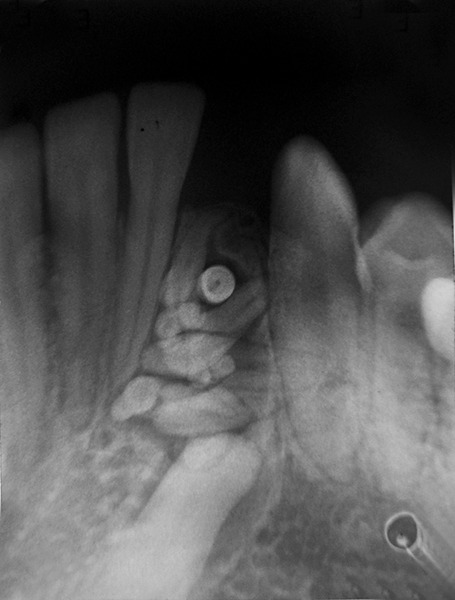
Radiograph of compound odontome

**Fig. 3:**
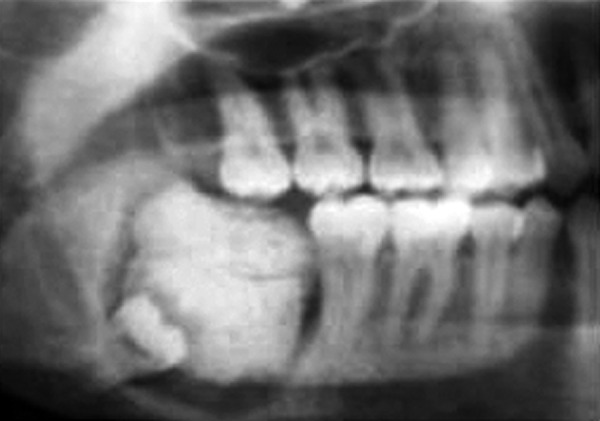
Radiograph of complex odontome

### Local Causes

The appearance of odontomes is liable to occur due to growth pressures because of inadequate space which have varying effects on the tooth development. Euler (1939)^23^ and Atkinson (1949)^24^ have both advanced opinion that these growth pressures may be of importance in some composite odontomes. This pressure theory was advanced by Hitchin and Ferguson(1958)^25^ that this might have arisen from a developing lower premolar germ of an inherited large crown form embraced by the roots of its deciduous predecessor, producing a pressure effect.

### Infection

Infection from the deciduous predecessor may also be a factor, though it is not likely to be early enough when derived from the deciduous predecessor; but a more generalized infection may be of some significance. The effects on the dentition of prenatal infection with treponema palladium, in children whose mothers have been infected with rubella during pregnancy, acute maxillitis of infancy, that acute pyogenic infection of the whole maxilla occurring shortly after birth in which, when examined at 61/2 years, was found to have a compound composite odontome. This was due to pyogenic infection causing division of a tooth germ.^23^ So in case of any infection, the occurrence of odontome may be due to the divison of a tooth germ or may interfere with tooth development. This may be pathologically related or affecting the genetic control of tooth development.

### Mature Ameloblasts

The etiology of odontomas is believed to have its origin from mature ameloblasts. Torreti et al^26^ suggested that these specialized cells have the potential of developing tumors with a wide variation in appearance and content.

### Cell Rests of Serres (Dental Lamina Remnants)

Fijerskov^27^ stated that cell rests of serres (dental lamina remnants) of the retained tooth with some epithelial island undergoing proliferation to develop into odontomes, while others underwent degeneration to form a cystic cavity enclosing the tooth for which the stimulus may be a genetic defect in the tooth forming process.

### Extraneous Odontogenic Epithelial Cells

They suggested that when these buds are divided into several particles they may develop individually to become numerous closely positional malformed teeth or tooth-like structures. When the buds develop without such uncommon division and consists of haphazard conglomerates of dental tissues, they may develop into complex odontome.^8,28-30^ However, the transition from one type to another is commonly associated with varying degree of morphodifferentiation or histodifferentiation or both, and it is often difficult to differentiate between both the types.^31^

### Trauma

Previous history of trauma has been implicated in production of the lesion, as has interference with the genetic control of tooth development, either inherited or due to mutation or due to extensive damage of the tooth germ.

Trauma to a developing tooth germ can also produce a hard tissue odontome. Andreasen (1994)^32^ describes an odontoma, like malformation of the permanent tooth germ, due to intrusive luxation or avulsion of the primary tooth. This malformation is rare sequela to injuries in primary dentition. The mechanism described by Andreasen is based on a history of a permanent tooth bud preemptive trauma. A vertically directed force through the long axis of the deciduous incisor was transmitted to the permanent tooth germ causing extensive damage. According to this theory, the malformation occurs during the early phase of odontogenesis and affects the morphogenetic stages of the ameloblastic development of the permanent tooth germ.^32^ Glasstone (1952)^23^ has shown that, if tooth germ of a rabbit is cut in half, each part develops in tissue culture into a complete rudimentary tooth, and Rushton (1957)^23^ has described a large nodule of enamel developing following trauma to a tooth germ before the completion of the enamel cap. Moreover, there are cases of odontome which appear to be due to the detachment of a portion of a tooth germ which may be from the epithelial sheath of Hertwig or from the enamel organ.^23^ In contradiction to the study by Hitchin, Levy^33^ showed that the pathological process is initiated only after amelogenesis has occurred. Levy produced a complex odontome by inducing trauma to developing first molars in rats. He further mentioned that whether trauma would produce hypoplasia, odontome or a supernumerary depended on the stage of development of the cells traumatized. In 1979, Shteyer, Taicher and Marmary^15^ reported a case of odontome occurring at the subcondylar region associated with a sinus tract linking to the third molar region. They deduced that the missing third molar had migrated to the subcondylar region followed by the occurrence of trauma or infection which led to the development of the odontome.

### Genetic Factors

The hypothesis regarding the etiology of the hard tissue odontome is that they are either inherited or due to a mutant or interference, possibly postnatal, with the genetic control of tooth development. Odontome can occur in one or more of three ways:

- By interference with the mechanism whereby genes control tooth formation and form
- By a mutation in the genes concerned
- By inheritance of those abnormal genes.

Hitchin suggested that a mutation in the epithelial cells of the tooth germ may change the inherent capacity of odontogenic epithelium to go through the cap and bell stages necessary for tooth formation, and yet retain its ability to stimulate mesenchymal differentiation necessary to form functional ameloblasts and odontoblasts, leading to the formation of an odontoma.^24^

Papagerakis et al^34^ suggested that the differentiation of normal and tumor odontogenic cells is accompanied by the expression of some molecules. The gene products present in some mesenchymal cells were also seen in the odontogenic tumor epithelium. The data may be related to a tumor-specific overexpression of the corresponding genes transcribed at an undetectable level during normal development and/ or to an epithelial-mesenchymal transition proposed to occur during normal root formation. A plausible explanation for the result is that odontogenic tumor epithelial cells are recapitulating genetic programs expressed during normal odontogeneis, but the tumor cells demonstrated abnormal expression patterns for these genes.

## SUMMARY

There are many unanswered questions about the origin of the odontomes. The occurrence is not necessary with one factor but it may be associated with multiple factors. It has been suggested that trauma and infection at the place of the lesion can offer ideal conditions for its appearance. This lesion is one of the odontogenic origin and is considered a self-limiting anomaly. Recently, Philipsen et al^10^ put forth the hypothesis that formation of a compound odontome is pathogenically related to the process producing hyperdon-tia, multiple schizodontia or locally conditioned activity of dental lamina.

So growth pressures, trauma, infection, mature am-eloblasts, cell rests of serres (dental lamina remnants), extraneous odontogenic epithelial cells may be regarded as sources of disturbances in the mechanism of development. They are also guided by mechanism where genes control tooth development.

## CLASSIFICATION

After Brocas (1866) first attempted to classify odontomas according to the stages of tooth development, many classifications were proposed according to structural tissues from which tumor arouse. ^1^

### WHO Classification

One of the most common classification is given by World Health Organization (WHO). Four lesions containing enamel and dentine of normal appearance are defined in the WHO classification.^35^ They are as follows:

1. *Ameloblastic fibro-odontome:* Consists of varying amounts of calcified dental tissue and dental papillalike tissue, the latter component resembling fibroma. The ameloblastic fibro-odontome is considered as an immature precursor of complex odontome
2. *Odonto-ameloblastoma:* Its a very rare neoplasm which resembles an ameloblastoma both structurally and clinically but contains enamel and dentine
3. *Complex odontome:* When the calcified dental tissues are simply arranged in an irregular mass bearing no morphologic similarity to rudimentary teeth
4. *Compound odontome:* Composed of all odontogenic tissues in an orderly pattern that results in many teeth-like structures but without morphologic resemblance to normal teeth.

On the basis of gross, radiographic and microscopic features^8,9,28,36^, two types of odontoma are recognized: (a) compound and (b) complex.

On the basis of their developmental origin^3,37^, in 1914, Gabell, James and Payne grouped odontome into three types:

- Epithelial
- Composite (epithelial and mesodermal) and
- Connective tissue.

According to their position within the jaws:^38,39^

- *Intraosseous (erupted odontoma):* They occur inside the bone and may erupt into the oral cavity. To date, 12 cases of the erupted variety have been described in the literature
- *Extraosseous or peripheral odontomas:* These are odontomas occurring in the soft tissue covering the tooth bearing portions of the jaws, having a tendency to exfoliate.

According to Thoma and Goldman (1946):^40,41^

- Germinated composite odontomes―two or more, more or less well-developed teeth fused together
- Compound composite odontomes―made up of more or less rudimentary teeth
- Complex composite odontomes―calcified structure, which bears no great resemblance to the normal anatomical arrangement of dental tissues
- Dilated odontomes―the crown or root part of tooth shows marked enlargement
- Cystic odontomes―an odontome that is normally encapsulated by fibrous connective.

Z Gorlin et al eliminated the term composite as redundant and classified odontomas as either complex or compound. There are essentially two types of odontome:^36,37^

- Complex composite odontome
- Compound composite odontome.

A new type known as hybrid odontome is also reported by some authors.

According to Robinson,^42^ in 1952, in his classification restricted the term odontome for those tumors which aroused from both epithelial and mesenchymal dental forming tissues. But presently, this term is used in a very restricted sense to designate only those tumors which consist of dental hard tissues.

Compound Odontome (Table 3)

These are the malformations in which all dental tissues are represented in a more orderly pattern, so that the lesion consists of many tooth like structures or denticles composed of enamel, dentin , cementum and pulp. It is a tumor of enamel and dentin arranged in the form of anomalous miniature teeth. Several small abnormal teeth surrounded by a fibrous sac.

Complex Odontome (Table 3)

These are the malformation in which all dental tissues are represented but not in an organized form or disorderly pattern. It is an odontogenic tumor characterized by the formation of calcified enamel and dentin in an abnormal arrangement because of lack of morphodifferentiation.

### Signs and Symptoms

Most of the odontomes are asymptomatic, although occasionally signs and symptoms relating to their presence do occur. These generally consist of unerupted or impacted teeth, retained deciduous teeth, swelling and evidence of infection.^5^ Compound odontomas seldom cause bony expansion but complex odontomes often cause slight or even marked bony expansion.^28,29^ The presence of odontomas may lead to malpositioning or displacement of adjacent teeth, aplasia, malformation and devitalization of adjacent teeth.^43^.

### Diagnosis

Majority of odontomes are diagnosed most commonly during routine radiographic examination. A developing odontoma can be detected by routine radiography but may cause difficulty in identification due to lack of calcification.^5^ A differential diagonosis is usually made through comparison of the degree of morphodifferentiation and histodifferentiation of the dental hard tissue.^3,28.^

A visual examination of the lesions cannot by itself define the differences between the complex and the compound types, because the odontomas are usually in the bone structures and do not show outward signs, such as expansion of the bone. Even in rare instances in which odontomas erupt into the oral cavity and can be examined visually and manually, the surface appearances of both types of odontoma are similar and differentiation between them is difficult.^31^ In comparison to visual examination and manual palpation, radiographic examination seems to be the most effective clinical method of discrimination between two types.^44^ In case of compound odontoma, radiographic image shows comparatively well-organized malformed teeth or tooth-like structures, usually is a radiolucent cyst like lesion. A complex odontoma shows an irregularly shaped oval radiopacity usually surrounded by a well-defined thin radiolucent zone. In case of compound odontoma in which extremely small, conglomerated malformed teeth or tooth-like structures are numerous, the radiographic image is similar to those obtained with complex odontomas and a differentiation between the two types may be difficult.^29,30^

So conventional radiography cannot always demonstrate details of difference. Because of poor image resolution, neither visual nor radiographic examinations showed the morphologic features necessary for diagnosis. To establish a definite diagnosis, some other procedure must be used; most commonly, histologic examination.^8,28,45^ However, because of the presence of transitions between types, even a histo-logic examination might not make a definite diagnosis possible.^31^ Microradiography is another useful procedure in the effort to establish a definite diagnosis. It enables histologic structures to be recognized from their various radiopacities and or radiolucencies. In addition, the resolution of microra-diography is equal to that obtained in histological examina-tion.^46^ Masayuki Kaneko (1998)^46^ et al showed in his case report that the low magnification radiograph showed the structural differences as clearly as did the histologic examination. High magnification microradiographs demonstrated even the finest dentinal tubules in detail, in correspondence with the histological images. So microradiographic findings showed that microradiographic images are not inferior to images obtained through hsitologic procedures.

## SUMMARY

The diagnosis of odontomas cannot be made by visual or manual techniques. It has to be done in coordination with radiographic as well as histological examination. The advance technique, such as microradiography, should be used to confirm the diagnosis.

## TREATMENT

Odontoma has a limited growth potential, but it should be removed because it contains various tooth formulations that can predispose to cystic change, interfere with eruption of permanent teeth and cause considerable destruction of bone.^40^ Because of the very low recurrence, the treatment of choice is surgical removal of the lesion. As it is a capsulated tumor, its removal is a simple surgical procedure but special care should be taken to remove it totally in order to avoid a relapse which is specially critical in immature complex odontomas. Odontomas are easily enucleated and adjacent teeth that may have been displaced are seldom harmed by surgical excision because they are usually separated by a septum of bone. But sometimes due to extension of the odon-tomes, the adjacent tooth may be disturbed while removal of the odontomes.^47^

A thorough visual, manual as well as radiographic examination should be performed for all the pediatric patients who present with clinical evidence of delayed eruption, missing tooth or temporary tooth displacement, with or without history of trauma. Early diagnosis of odontomas helps us to:

1. Adopt a less complex and less expensive treatment
2. Ensures better prognosis
3. Avoid relapse of the lesion
4. Avoid displacement or devitalization of adjacent tooth.
